# Molecular diagnostics in paediatric oncology in Latin America: findings from the Latin America Regional Advisory Committee (RAC LATAM) regional assessment

**DOI:** 10.3332/ecancer.2026.2157

**Published:** 2026-06-26

**Authors:** Julia Palma, Sofía Aljaro, Daniela Arce, Milena Villarroel, Federico Antillón, Luiz Lopes, Nataly Mercado, Adriana Morais, Andrés Portilla, Leonardo Arana, Guillermo Chantada, Mónica Cypriano, Soad Fuentes, Augusto Pereira, Lourdes Vega, Nubia Zuñiga, Liliana Vásquez, Andrea Capellano, Paola Friedrich

**Affiliations:** 1 RAC LATAM Molecular Biology Working Group, St. Jude Global, Memphis, TN 38105, USA; 2 RAC LATAM, St. Jude Global, Memphis, TN 38105, USA; 3 PAHO Washington, Washington, DC 20037, USA; 4 SLAOP (Sociedad Latinoamericana de Oncología Pediátrica A.C.), Puebla 72530, Mexico

**Keywords:** molecular biology, paediatrics, neoplasms, diagnosis, health equity

## Abstract

Molecular diagnostics are an essential component of paediatric oncology care, providing critical information for disease classification, risk stratification and the selection of targeted therapies, ultimately improving patient outcomes. This study presents the results of the 2024 Latin America Regional Advisory Committee and Paediatric Oncology Latin American Society regional survey, which assessed the availability, accessibility, quality and key challenges associated with molecular diagnostics for paediatric malignancies across Latin America. While molecular testing is available in several countries, access remains heterogeneous and inconsistent, particularly in public healthcare settings. Survey findings highlight significant barriers, including financial limitations, a shortage of trained personnel and insufficient laboratory infrastructure. The study also explores potential strategies to strengthen regional capacity through international collaborations, sustainable financing models and targeted workforce development initiatives, to promote equitable access to precision diagnostics for all children with cancer in the region.

## Background

There is robust evidence in the medical literature that molecular diagnostics have significantly transformed paediatric oncology by enabling more precise tumour classification and facilitating the use of targeted therapies. Next-generation sequencing (NGS) and other molecular profiling techniques have led to the identification of actionable mutations and the reclassification of tumours, directly impacting diagnostic accuracy and therapeutic decision-making [1]. However, significant disparities remain in the access, interpretation and application of these technologies in low- and middle-income countries (LMICs), particularly in Latin America (LATAM) [2]. This work compiles regional experiences, perspectives and recent developments in paediatric oncogenomics, with a focus on the implementation of technologies such as NGS, deoxyribonucleic acid methylation and transcriptomics. It also highlights the importance of collaborative networks and the establishment of specialised biobanks.

Molecular diagnostics have revolutionised paediatric oncology care by enabling more accurate classification and targeted treatment of malignancies [3, 4]. However, access to these technologies remains uneven across LMICs [5]. This study assesses the availability, infrastructure and main challenges related to molecular diagnostics for paediatric malignancies in LATAM, based on survey responses from Paediatric Oncology Latin American Society (SLAOP) country delegates.

Reference centers in Argentina, Brazil, Chile, Mexico, Peru and the United States – such as Argentinean Centro de Educación Médica e Investigaciones Clínicas ‘Norberto Quirno’, Mexican Instituto Nacional de Medicina Genómica (INMEGEN), Peruvian Instituto Nacional de Enfermedades Neoplásicas (INEN), St. Jude Children’s Research Hospital and Chilean Calvo Mackenna Hospital – have spearheaded initiatives in genomic sequencing and molecular classification of paediatric solid and hematologic tumours.

Despite regional disparities, several countries presented promising initiatives. Peru highlighted the National Tumour Bank at INEN [6], with over 20,000 samples and plans for a national cancer genomics biobank. Brazil presented research on paediatric germ cell tumours [7, 8] and evaluated Nanopore sequencing as a low-cost diagnostic alternative. Mexico showcased two major efforts: Hospital Infantil Teletón de Oncología high-resolution NGS diagnostics for leukaemia and solid tumours, and INMEGEN’s integration of NGS [9] and pharmacogenomics in public healthcare, supported by public–private partnerships.

Chile shared its transition toward precision medicine at Calvo Mackenna Hospital, emphasising hereditary cancer predisposition and the creation of an oncogenetics clinic. Argentina presented two key initiatives: the Colaboración en Oncología Pediátrica de Precisión de Argentina project, offering free genomic profiling and a regional interpretation network and population-specific genomic analysis to enhance precision oncology, contributing to global databases. These efforts reflect the region’s commitment to building local genomic capacity, training specialised professionals and fostering sustainable collaborations to improve paediatric cancer care.

Beyond technical aspects, the shared regional experiences underscore a growing consensus on the need for clinical professionals trained in genomics, adequate bioinformatics infrastructure and solid ethical and logistical frameworks [10]. Furthermore, the generation of local genomic data is crucial for accurate variant interpretation and to address the current underrepresentation of Latin American populations in global genomic research.

Together, these initiatives showcase both achievements and ongoing challenges in the effort to build an equitable, context-aware model of precision oncology in LATAM, positioning genomics as a key tool to reduce diagnostic gaps in paediatric cancer.

Despite ﻿scientific and technological advances in genomic medicine, its effective implementation in LMICs remains limited. This creates a critical gap in early diagnosis, risk stratification and personalised therapy selection for paediatric cancer, with direct consequences on survival and quality of life outcomes. Diagnostic platforms developed in high-income countries are not always viable or sustainable in other contexts, underscoring the need for innovative, collaborative strategies tailored to local needs. Growing evidence supporting the role of molecular characterisation in both leukaemias and paediatric solid tumours reinforces the shift toward more integrated, accessible and clinically meaningful diagnostic models.

Additionally, the identification of population-specific genetic variants in Latin American populations highlights the importance of generating local genomic knowledge for accurate and contextualised interpretation. These efforts not only enhance diagnosis and therapeutic approaches but also strengthen scientific sovereignty and advance health equity across the region.

This study aims to :

1. Characterise current strategies for genomic implementation in paediatric cancer across reference centers in LATAM, as well as through international collaborators.
2. Identify common barriers, facilitators and enabling factors for the integration of advanced genomic tools, including technical, human resource, financial and organisational dimensions.
3. Showcase successful experiences in generating local genomic data, biobanking and integrated analysis platforms as essential components for precision medicine.

This effort seeks to inform public policies, guide capacity-building initiatives and foster collaborative frameworks that promote more accessible, effective and locally driven genomic medicine for children with cancer in LATAM.

## Methods

This was a cross-sectional, descriptive study conducted via an online survey to assess the availability, accessibility and implementation challenges of molecular diagnostics for paediatric oncology across LATAM. A structured questionnaire was designed by experts from the Latin America Regional Advisory Committee (RAC LATAM)/SLAOP network and included both closed- and open-ended questions. The survey was developed to collect institutional-level data and was divided into the following thematic sections:

- General information (country, institution type and respondent role)
- Availability of molecular tests (e.g., Polymerase Chain Reaction (PCR), fluorescence *in situ* hybridisation (FISH), NGS and karyotyping)
- Access and infrastructure (in public/private settings)
- Quality assurance (accreditation, turnaround times)
- Barriers to implementation (funding, equipment and trained personnel)
- International collaborations and future needs

The survey was distributed between March and June 2024. Dissemination occurred via the RAC LATAM/SLAOP coordination team using national contacts. Institutions were asked to designate one respondent (clinical lead or laboratory specialist). Responses were submitted through an online platform and collected centrally for analysis. A total of 17 institutions from multiple Latin American countries participated. Institutions represented both public and private paediatric oncology centers. Quantitative responses were summarised using descriptive statistics. Qualitative responses were reviewed thematically to extract insights on barriers, needs and proposed solutions.

## Results

1. General information

Seventeen countries participated in the survey. Represented institutions ranged from national cancer institutes to paediatric hospitals. 70.6% were public institutions, 17.6% were private and 11.8% were mixed. Results by institution are shown in Table 1, including countries that responded to the questionnaire, social category in which the test is made, possibility or not of performing molecular diagnosis, need for international shipping, number of centers per country and type of methods available for diagnosis.

2. Availability of molecular studies

- When asked whether there were molecular tests for paediatric neoplasms, of the 17 countries that responded, 14 (76.5%) said yes. The same number of institutions answer that they have the possibility of sending samples to another country.
- Of those who have access to molecular tests, 78.6% have less than 5 centers that provide this service, 14.3% have between 5 and 10 and 7.1% have more than 20.
- The PCR technique was the most available among the 17 countries (93.3%), followed by FISH (66.7%) and NGS (53.3%). Other and more sophisticated techniques were available in few countries.
- 93.3% of the countries have access to this type of test for acute lymphoblastic leukaemia, 80% for acute myeloid leukaemia and 53.3% for Ewing Sarcoma.
- The turnaround time for receiving results is variable. 40% receives them in 1–2 weeks and another 40% receives them in 2–4 weeks. In 13.3% of the institutions takes more than 4 weeks and only one (6.7%) has the results in less than 1 week.
- Some countries rely on international laboratories for complex molecular studies due to infrastructure and resource limitations.
- Molecular diagnostics are also used for adult malignancies in certain institutions.

3. Accessibility and infrastructure

- Sixteen countries answered which type of institutions perform molecular testing locally. 50% count on public hospitals, 62.5% on private institutions, 50% independent commercial laboratories and 25% on academic or university laboratories. The cost of these tests is fully covered by national health insurance on 25% of the countries (Colombia, Perú, Argentina and Costa Rica) and partially covered in 50%.
- Eleven countries responded to the average cost when not covered by health insurance. For 45.5%, the average cost in US dollars was less than 500 dollars, 27.3% between $500 and $1,000, 18.2% between 1,000 and 2,500 and 9.1% have to pay more than $2,500.
- Only 37.5% of the countries that answered the survey state that more than 75% of paediatric oncology patients have access to molecular diagnosis, 18.8% covers between 25% and 50% and 43.8% refers to access to less than 25% of them.

4. Quality and training

- Only four countries (Colombia, Ecuador, Mexico and Chile) have a national quality assurance program for molecular diagnosis in paediatric oncology.
- 47.1% reported that paediatric oncologists in their country have access to training in this area. Additionally, 58.8% felt comfortable interpreting and applying these tests, 23.5% felt neutral and 17.7% did not feel comfortable at all.
- 58.8% of the countries have collaborations with international institutions.

5. Challenges and opportunities

- Regarding the primary challenges in implementation of these techniques, 70.6% mentions the limited availability of technology or infrastructure, 64.7% the limited access to certain types of molecular tests and lack of funding. 52.9% allude to the lack of trained personnel as one of the main challenges their country face.
- Some steps that are taken to face these challenges are, for example, the development of laboratories of genetics and molecular biology in cancer institutes as well as children’s health institutes in Peru. These facilities are staffed with medical geneticists and molecular biologists who are implementing these advancements. Panama mentions the training of personnel (pathologists and technicians), acquisition of equipment (NGS) and establishing conversations with distribution companies.

## Discussion

This study provides the first comprehensive situational analysis of the availability, accessibility and quality of molecular diagnostics for paediatric and adolescent cancer across LATAM. The findings underscore a stark disparity in access to timely and high-quality molecular testing, which remains essential for accurate diagnosis, risk stratification and the delivery of precision medicine in paediatric oncology.

While most surveyed countries reported some level of molecular diagnostic capability, these services are unevenly distributed. Basic techniques such as PCR and FISH are more widely accessible, particularly for leukaemias, but advanced methods like NGS are limited to a few private centers or require international outsourcing. This creates inequities that contradict the principle of universal health coverage, particularly as molecular diagnostics are increasingly recognised as a standard of care in high-income settings.

The analysis also highlights that public healthcare systems, which serve most of the population in LATAM, face significant barriers. These include limited reimbursement policies, inadequate infrastructure, lack of trained personnel and delays due to logistical challenges in sample transport. This fragmentation not only compromises timely diagnosis and treatment but also exacerbates survival gaps between countries and within populations.

Another critical gap lies in the integration of molecular data into clinical practice. While some centers benefit from trained geneticists and access to international collaborations, many institutions lack local training programs, standardisation protocols and genetic counseling services. This hinders the translation of molecular insights into clinical decisions and family care, particularly in hereditary cancer syndromes.

Importantly, the review of National Cancer Control Plans (NCCPs) revealed that molecular diagnostics are rarely incorporated into national strategies, signaling a missed opportunity to institutionalise these tools in public health frameworks. The reluctance of some governments to adopt molecular testing – due to concerns about associated costs and the need for targeted therapies – further delays progress, despite growing evidence of cost-effectiveness and clinical benefit. Advocating for standardised molecular diagnostic panels and policy integration into NCCPs might be the next step, exchanging best practices across ministries and national teams.

In many countries, foundations that raise funds to support the diagnosis and treatment of paediatric and adolescent cancer either provide the funds to support molecular diagnosis or provide the service itself. These initiatives highlight the importance of local Foundations in the effort to provide curative treatment for paediatric cancer.

Although the information presented in this study provides a valuable snapshot of the regional landscape, it is primarily based on survey responses from SLAOP country delegates. While these inputs offer important insights, they are not exhaustive and may not fully capture the diversity and complexity of the situation within each country. Therefore, more detailed, granular and systematically collected data are necessary to draw robust conclusions and to guide effective, country-specific policy and implementation strategies.

Despite these limitations, the data collected allows the identification of key entry points for regional action. Strategies based on current findings may include:

- Mapping and strengthening existing molecular diagnostic hubs to serve as reference centers for high-complexity testing
- Developing national guidelines for molecular diagnostics in paediatric oncology, integrating molecular diagnostics into NCCPs and Universal Health Coverage frameworks
- Explore the role of local Foundations in supporting more extended molecular diagnosis
- Promoting regional training programs and professional exchanges to address the shortage of specialised personnel
- Creating collaborative frameworks for public–private partnerships to fund infrastructure and testing access. These approaches can be gradually implemented, adapted to national contexts and aligned with regional priorities to foster equity and sustainability.

Another critical outcome of this study is the identification of key areas where support and resources are most needed:

- Funding: To cover the cost of tests, reagents, laboratory infrastructure and logistical processes.
- Training: For both laboratory personnel and clinicians in the interpretation and clinical application of molecular results.
- Equipment: To build and expand in-country molecular diagnostic capacity.
- Insurance and coverage reform: To ensure molecular diagnostics are incorporated into public health benefit packages and reimbursed by national insurance schemes.
- International collaboration: To facilitate access to technical assistance, capacity building, shared platforms and knowledge transfer through regional and global partnerships.

The Coronavirus disease (COVID-19) pandemic further exposed and deepened structural weaknesses in diagnostic access across the region. Many institutions reported major disruptions in essential services – such as laboratory testing, imaging and pathology, especially in lower-income countries. Global data showed that routine diagnostic activity decreased sharply, with a 43% drop in new paediatric cancer diagnoses reported in the first year of the pandemic. Limited infrastructure, fragile supply chains and the reallocation of health resources worsened delays and made it even more difficult to access specialised tests, including molecular diagnostics [11].

Although some hospitals adopted alternative strategies – such as telemedicine or prioritisation protocols – these responses could not fully compensate for the reduced diagnostic capacity. Even now, long after the peak of the pandemic, many barriers remain unresolved. Recovery has been slow and uneven, and some institutions continue to face shortages of supplies, staff and funding.

Strengthening molecular diagnostic capacity should therefore be seen not only as a way to prepare for future health emergencies but also as a response to the chronic instability and underinvestment that affect many health systems in LATAM. Building sustainable access will require coordinated action among governments, health institutions, scientific societies and civil society. Long-term financing, shared governance models, workforce development and interoperable health systems will be essential to achieve this goal.

A phased, collaborative approach is key. Centralising advanced testing in reference laboratories while ensuring local capacity for clinical interpretation and care, the so-called ‘hub-and-spoke’ model, may offer a practical way forward. It is also urgent to map regional capabilities, implement quality assurance systems and invest in regional and cross-border partnerships. These efforts will be critical not only for reducing disparities but also for making the benefits of precision medicine available to all children with cancer in the region.

Finally, there is an urgent need to map existing regional capacities, development of quality assurance mechanisms and investment in national and cross-border partnerships. These steps are crucial not only for reducing disparities but to harness the full potential of precision medicine for all children with cancer across LATAM.

## Conclusion

The RAC LATAM/SLAOP 2024 survey underscores the urgent need to strengthen infrastructure, mobilise financial resources and foster regional collaboration to ensure equitable access to molecular diagnostics for paediatric malignancies in LATAM.

## Conflicts of interest

The authors declare that they have no conflicts of interest.

## Funding

The authors received no financial support for the research, authorship and/or publication of this article.

## Author contributions

All authors contributed equally to the conception, development and writing of the manuscript.

## Figures and Tables

**Table 1.** Institutional characteristics and availability of molecular diagnostic methods by country.

Country	Type	Molecular diagnosis	International shipping	N° centers	Tests availability
Argentina	Public	Yes	Yes	< 5	NGS, FISH, PCR, RNA Seq, Sanger Seq
Bolivia	Public	Yes	Yes	< 5	FISH, PCR
Brazil	Public	Yes	No	> 20	NGS, FISH, PCR, WES, RNA Seq, Sanger Seq, GEP, Medulloblastoma
Chile	Public	Yes	Yes	< 5	NGS, FISH, PCR
Colombia	Private	Yes	Yes	5–10	NGS, CMA, FISH, PCR, WES, RNA Seq, GEP
Costa Rica	Public	Yes	Yes	< 5	NGS, CMA, FISH, PCR, WES, RNA Seq, Sanger Seq, GEP
Ecuador	Public	Yes	No	< 5	NGS, FISH, PCR
Guatemala	Mixed	Yes	Yes	< 5	PCR
Honduras	Public	No	Yes	-	-
Mexico	Public	Yes	No	-	PCR
Nicaragua	Public	No	No	-	-
Panama	Public	No	Yes	< 5	NGS, CMA, PCR
Paraguay	Private	Yes	Yes	< 5	CMA, FISH, PCR
Peru	Public	Yes	Yes	< 5	PCR, Sanger Seq, Cytogenetics
Dominican Rep.	Public	No	Yes	< 5	-
Uruguay	Public	Yes	Yes	< 5	NGS, CMA, FISH, PCR
Venezuela	Private	Yes	Yes	5–10	CMA, FISH, PCR
NGS, Next generation sequencing; FISH, Fluorescence *in situ* hybridisation; PCR, Polymerase chain reaction; RNA seq, RNA sequencing; Sanger Seq, Sanger sequencing; CMA, Chromosome microarray; WES, Whole exome sequencing; GEP, Gene expression profiling. All techniques used in molecular biology and genetics					
